# Net Effects of Ecotourism on Threatened Species Survival

**DOI:** 10.1371/journal.pone.0147988

**Published:** 2016-02-17

**Authors:** Ralf C. Buckley, Clare Morrison, J. Guy Castley

**Affiliations:** School of Environment, Griffith University, Gold Coast, Australia; Università degli Studi di Napoli Federico II, ITALY

## Abstract

Many threatened species rely on ecotourism for conservation funding, but simultaneously suffer direct ecological impacts from ecotourism. For a range of IUCN-Redlisted terrestrial and marine bird and mammal species worldwide, we use population viability analyses to calculate the net effects of ecotourism on expected time to extinction, in the presence of other anthropogenic threats such as poaching, primary industries and habitat loss. Species for which these calculations are currently possible, for one or more subpopulations, include: orangutan, hoolock gibbon, golden lion tamarin, cheetah, African wild dog, New Zealand sealion, great green macaw, Egyptian vulture, and African penguin. For some but not all of these species, tourism can extend expected survival time, i.e., benefits outweigh impacts. Precise outcomes depend strongly on population parameters and starting sizes, predation, and ecotourism scale and mechanisms. Tourism does not currently overcome other major conservation threats associated with natural resource extractive industries. Similar calculations for other threatened species are currently limited by lack of basic population data.

## Introduction

A primary goal of conservation is to maintain biological diversity, principally by minimising species extinctions [1–7]. Public protected areas provide the main mechanism, but conservation measures on other public, private and communal lands are increasingly important [2,3,8–13]. Extinctions continue despite all these measures [1–9]. Shortage of funds is a major barrier to each [9–15], and conservation on all land tenures relies increasingly on ecotourism for financial and political support [14–18]. National parks agencies worldwide receive up to 84% of funding from ecotourism [19], and for the 360 threatened mammal, bird and frog species for which data are available, ecotourism funds conservation of up to 66% of remaining individuals [19–20] and up to 99% of remaining habitats [21]. Ecotourism also generates ecological impacts, however, as shown for over 800 species [22]. As ecotourism is used and advocated more widely in conservation [15–18,23–25], quantifying its net outcomes has become correspondingly urgent.

There are few previous attempts to calculate the net conservation consequences of ecotourism benefits and impacts simultaneously, and all suffer significant shortcomings. There are small-scale case studies linking tourism and conservation for individual species and sites [17,23–26]. There is one national-scale study, in Costa Rica, which examines the net effects of ecotourism [14]. Its focus, however, was on poverty rather than threatened species, and it used a very indirect measure of ecotourism, the presence of a park entrance within a census tract. In conservation practice, ecotourism is in wide and rapidly increasing use; but in conservation theory, attempts to measure outcomes have been localized or indirect. Here, therefore, we apply best available ecological models to quantify the effects of real-world ecotourism interventions in threatened species conservation. Our focus is on cases where both the positive and negative effects of ecotourism can be identified and quantified simultaneously for the same species and subpopulations. There are also many other cases, both within and outside public protected areas, where earnings from ecotourism are not used for conservation, but those are not our focus here.

The most direct, relevant and immediate measure for the conservation outcome of any human intervention is a change in the expected time to extinction for individual threatened species. The most precise and reliable tool to calculate this change is population viability analysis, PVA, which has been designed and refined specifically for that purpose [27–29]. PVA relies on detailed empirically-measured population parameters. It achieves reliability through sensitivity analysis and by averaging results from repeated iterations. PVA has the imperfections of all predictive mathematical models, but it provides the current gold standard in evaluating conservation of threatened species, and has been applied in many individual conservation interventions [30–33].

Here we apply PVA to determine the net population consequences of ecotourism for those individual threatened species where all relevant parameters are available. The PVA approach measures the consequences of ecotourism directly against the goals of conservation, and thus represents a significant advance in relevance and reliability over previous analyses. To our knowledge, this has not been attempted previously, because ecotourism benefits and impacts have been calculated for different species, at different scales, using different parameters and units [14,18–22]. We overcome these difficulties by converting each individual effect of ecotourism into changes in population parameters such as habitat area, reproductive rate, or juvenile or adult mortality, for each species. We show that even within this restricted species set, net outcomes of ecotourism differ between species, sites and subpopulations. In deploying ecotourism for conservation, this approach can predict when, where and whether particular patterns in ecotourism will yield net gains for individual threatened species.

## Materials and Methods

Population viability analyses are discrete mathematical models which track and predict the population sizes and structures of individual species, one generation at a time [27–29]. They rely on data on the size, sex ratio, mortality, fecundity, and inward and outward dispersal of each age class in each subpopulation. These parameters must be measured empirically. More recent algorithms have improved reliability and predictive accuracy. Since they are iterative, however, they remain sensitive to small changes in the numerical values of starting population parameters [28]. To calculate the net outcomes of ecotourism on threatened species, its conservation funding effects and ecological impacts must first be converted to the population parameters required for PVAs. These effects operate through a variety of different processes, at a range of different scales.

Effects operating through provision of financial and political support for conservation have been examined mainly at large scale. To express these effects as population parameters for threatened species requires scaling down, to assign ecotourism funding to specific conservation actions. There are three main processes involved. Populations of individual species, in all age classes, are increased by funding the acquisition or allocation of additional habitat areas, and restoration or modification of habitat to make it more suitable for particular species. Mortality of individual species, in all age classes, is reduced by funding: anti-poaching patrols and equipment; local community conservation-incentive and compensation programs; live-capture and veterinary services; and supplementary feeding programs, including the introduction of prey species. Fecundity and recruitment of individual species is increased directly by funding captive breeding programs, and indirectly by funding translocation programs, which modify sex ratios, age-class structures and genetic diversity in individual subpopulations, and reduce mortality from territorial aggression.

Ecological impacts of ecotourism on threatened species have previously been studied principally at small scale, on local subpopulations [22]. These local-scale impacts of ecotourism modify population parameters as follows. Populations in all age classes are reduced by loss and degradation of habitat, through the development of tourism infrastructure. Mortality, both juvenile and adult, is increased through a wide variety of species-specific processes. Examples include: direct impact by cars or boats; sport hunting and souvenir collection; repeated foraging interruptions during energy-limited migration or overwintering; and human disturbance, loss of vigilance, and consequent increased predation. Fecundity and recruitment are reduced through direct disturbance to courtship and reproductive behaviors; disruption and displacement from preferred breeding sites; increased predation and accidental death of offspring through human disturbance; and various indirect effects [22].

To calculate the net effects of ecotourism, we identified threatened species which satisfy four criteria. Firstly, the species has been subject to population viability analysis (PVA), published since 2000. Earlier PVAs were excluded since PVA algorithms, and the population parameters required as inputs, have evolved over time, so that pre-2000 publications generally do not include the information needed to re-run the PVAs using current algorithms [27]. We identified 133 species meeting the first criterion. Secondly, the species acts as an attraction for ecotourism. We identified 64 species meeting both the first and second criteria. Thirdly, there is also independently published quantitative information on both conservation funding effects and ecological impacts of ecotourism, which can be expressed as the population parameters used in current PVA algorithms. We identified 20 species meeting all three of these criteria. Fourthly, the published PVAs contain all the population parameters required to repeat the analysis and reproduce its original results. Only nine species met all four criteria. Eleven species, including bison, hippopotamus, western gorilla, and proboscis monkey, met the first three but not the fourth, despite our efforts to obtain missing data from the original authors of each published PVA.

For each species satisfying all four criteria, we re-ran the original published PVAs using VORTEX 9.99, the most commonly used PVA software [27], incorporating all ecotourism effects simultaneously. We obtained average predicted population trajectories for individual threatened species and subpopulations by re-running each PVA, incorporating the simultaneous effects of direct ecotourism impacts, and ecotourism funding for conservation, at three different ecotourism intensities, plus the baseline without ecotourism. Where applicable, we also included other local anthropogenic impacts, unrelated to ecotourism. We compared different baseline starting populations, following the original published PVAs for each species. The conversion of ecotourism effects to population parameters differs between species (Tables 1–3). To check that the reliability of predictions is robust to small variations in these conversions, we conducted sensitivity analyses for each conversion from ecotourism effect to population parameter. The detailed parameters, outputs and sensitivity analyses for the population viability analyses are listed in Tables 1–3. We ran each analysis for the same number of iterations (100–1000), and the same time period, as originally published. We then compared the outcomes for each species at the year 2050, a relatively long time horizon in practical conservation planning.

**Table 1:** Effects of ecotourism activities and impacts on species population parameters.

Activity *(& References)*	Impacts	CC	AS	AB	SG	RR	JS
**Largely Negative**							
Infrastructure development [34,35]	Habitat loss, loss of individuals, loss of prey or forage	-	-			-	
Sport hunting [36]	Loss of individuals, altered behaviour		-	+/-		+/-	
Motorised activities [35,37]	Habitat loss or modification, altered behaviour, loss of individuals	-	-			-	
Direct disturbance [35, 37–41]	Interrupt foraging, interfere with territorial and courtship behaviour, increase predation on juveniles			-		-	-
Introduction alien species [42]	Increased predation, competition, disease, habitat modification		-			-	
**Largely Positive**							
Habitat expansion [21,43,44]	Opportunities for population growth, corridors for migration, exchange	+	+	+	+	+	+
Habitat restoration [35,45]	Improve habitat quality	+					
Remove alien predators and invasives, control fire [43,45,46]	Improve habitat quality, reduce mortality	+	+			+	+
Habitat protection (e.g. fencing), visitor management [35,39,41,47]	Reduce outside access, reduce conflicts with local communities, reduce trampling, reduce access to vulnerable sites e.g. breeding sites		+			+	+
Reintroductions and translocations of individuals, breeding programs, veterinary services [36,42,47–49]	Increase population size, increase genetic diversity, increase prey availability	+			+	+	
Anti-poaching patrols [35,42,47]	Reduce hunting pressure		+				
Supplementary feeding, introduce prey species [34,36]	Reduced mortality	+			+	+	+

CC, carrying capacity; AS, adult survival; AB, adults breeding; SG, age structure and/or genetic diversity; RR, reproductive rate; JS, juvenile survival.

**Table 2:** Changes in 5 key population parameters for each species at different ecotourism intensities.

Species *(References)* and Sites	IUCN Category	Tourism	CC %	AP %	AS %	AB %	JS %
Cheetah [36]	VU	Low			+1	+5^1^	+1
Southern Africa		Med	+0.7^2^		+2	+10^1^	+2
		High	+1.5^2^		+5	+10^1^	+0
Vulture [34,50]	EN	Low			+10		+10
Spain		Med			+20		+20
		High			+30		+30
Tamarin [42]	EN	Low	+1	+0.02	-1		-1
Brazil		Med	+5	+0.06	-2		-2
		High	+10	+0.08	-5		-5
Macaw [43,44]	EN	Low	+1				+2
Costa Rica		Med	+2				+5
		High	+5				+10
Gibbon [41]	EN	Low	+5				
India		Med	+10				
		High	+15		-2		
Penguin [45,46,51–54]	EN	Low			-1		+2
Southern Africa		Med			-5		+7
		High			-10		+15
Orangutan [35,47]	CR	Low	+1	+0.2			
Sumatra		Med	+5	+0.4			
		High	+7	+0.8			
Sealion [37,40,55]	EN	Low					-1
New Zealand		Med					-5
		High					-10
Wild dog [48,49]	EN	Low	+5	+5^3^			
Southern Africa		Med	+10	+10^3^			
		High	+15	+20^3^			

IUCN Categories: VU, Vulnerable; EN, Endangered; CR, Critically Endangered. CC, carrying capacity; AP, adult population; AS, adult survival, all adult age classes; AB, adults breeding; JS, juvenile survival.

^1^, breeding females.

^2^, reintroductions.

^3^, annual exchanges between subpopulations, no net addition.

+, increase in parameter.

-, decrease in parameter.

Figures show percentage changes for each. Full species names given in Table 3.

**Table 3:** PVA predictions for different starting populations and tourism intensities.

Species *(& References)*	Y/i/N_i_	Tourism	r ± SD	PE	N ± SE	TE
Cheetah	100/500/150	None	0.104 ± 0.184	0.000	125 ± 24	0.0
*Acinonyx jubatus*^*1*^	(also 80, 400)	Low	0.131 ± 0.184	0.000	128 ± 23	0.0
[36]		Med	0.170 ± 0.181	0.000	136 ± 23	0.0
		High	0.225 ± 0.174	0.000	142 ± 25	0.0
Egyptian vulture	50/200/470	None	0.039 ± 0.122	0.000	1141 ± 110	0.0
*Neophron percnopterus*^*1*^	(also 116, 322,	Low	0.049 ± 0.118	0.000	1177 ± 89	0.0
[34]	544, 608)	Med	0.061 ± 0.111	0.000	1215 ± 55	0.0
		High	0.070 ± 0.106	0.000	1235 ± 33	0.0
Golden lion tamarin	100/500/200	None	0.057 ± 0.160	0.006	159 ± 37	32.3
*Leontopithecus rosalia*		Low	0.052 ± 0.155	0.000	157 ± 34	51.5
[42]		Med	0.057 ± 0.154	0.000	180 ± 30	0.0
		High	0.040 ± 0.148	0.000	195 ± 40	69.0
	100/500/40	None	0.071 ± 0.138	0.012	32 ± 7	84.3
	(also 10)	Low	0.089 ± 0.141	0.000	35 ± 6	0.0
		Med	0.111 ± 0.148	0.000	39 ± 6	35.0
		High	0.090 ± 0.155	0.000	43 ± 7	0.0
Great green macaw	100/500/210	None	0.036 ± 0.088	0.000	293 ± 12	0.0
*Ara ambiguus*	(also 80*, 350*)	Low	0.039 ± 0.087	0.000	298 ± 12	0.0
[44]		Med	0.043 ± 0.085	0.000	315 ± 9	0.0
		High	0.050 ± 0.082	0.000	346 ± 9	0.0
Hoolock gibbon	100/500/50	None	0.006 ± 0.058	0.004	35 ± 9	90.0
*Hoolock hoolock*	(also 100*, 200)	Low	0.007 ± 0.054	0.004	41 ± 10	90.5
[41]		Med	0.008 ± 0.052	0.004	47 ± 10	91.5
		High	0.012 ± 0.049	0.000	50 ± 7	0.0
African penguin	25/100/2240	None	-0.040 ± 0.112	0.000	857 ± 250	0.0
*Spheniscus demersus*		Low	-0.039 ± 0.112	0.000	883 ± 234	0.0
[45,46,54]		Med	-0.035 ± 0.111	0.000	971 ± 253	0.0
		High	-0.012 ± 0.105	0.000	1708 ± 438	0.0
	25/100/4480	None	0.057 ± 0.104	0.000	14602 ± 1966	0.0
		Low	0.056 ± 0.107	0.000	14569 ± 2262	0.0
		Med	0.056 ± 0.107	0.000	14690 ± 2226	0.0
		High	0.062 ± 0.101	0.000	15698 ± 1896	0.0
Orangutan	1000/500/500	None	0.003 ± 0.049	1.000	0 ± 0	21.3
*Pongo abelii*^*1*^	(also 50, 1000)	Low	0.014 ± 0.064	1.000	0 ± 0	26.3
[35]		Med	0.013 ± 0.052	0.000	484 ± 16	0.0
		High	0.004 ± 0.032	0.000	4237 ± 207	0.0
Sea lion	100/500/11200	None	-0.010 ± 0.051	0.206	7421 ± 5790	86.1
*Phocarctos hookeri*	(also 2000*,	Low	-0.015 ± 0.052	0.330	5681 ± 4645	85.1
[37,55]	6500*	Med	-0.026 ± 0.054	0.768	3041 ± 2031	78.7
		High	-0.037 ± 0.055	0.990	1374 ± 509	64.3
Wild dog	100/1000/8	None	0.175 ± 0.398	0.142	15 ± 7	15.2
*Lycaon pictus*	(also 15*, 25*)	Low	0.221 ± 0.398	0.002	20 ± 4	26.2
[48,49]		Med	0.231 ± 0.398	0.000	23 ± 4	35.7
		High	0.310 ± 0.373	0.000	29 ± 3	55.2

Y, number of years models run; i, number of iterations; N_i_, initial population size; r, mean annual rate of population change; PE, probability of extinction; N, mean final population size; TE, mean time to extinction. Tourism = None, baseline models as per source referenced; Low, Med, High, parameters as per Table 2.

^*1*^ Statistics shown for selected N_i_ only; results for additional N_i_, shown as “(also as N_i1_,N_i2_,…)” in column 2, were similar.

*, additional N_i_ added by authors of this study; all other N_i_ are from PVAs as originally published.

## Results

The practical mechanisms by which threatened species receive support from ecotourism (Table 1) include: funding to public national parks [19–21,24], establishment of private reserves [17,25,56], switching communal land use from consumption to conservation [15,24,57], and funding of breeding, feeding, translocation, veterinary, anti-poaching, livestock-compensation and conservation-incentive programs [24,25,57,58]. Mechanisms by which threatened species suffer impacts from ecotourism (Table 2) include changes to plant and animal physiology, nutrition, pathology, reproduction, individual mortality, populations and habitat [22]. We include all these mechanisms as they apply to each individual species.

We identified 133 threatened species worldwide, including 122 mammals and birds, with PVAs published since 2000. Relevant population parameters are lacking for other threatened species influenced by ecotourism [59,60]. Such data deficiencies are widespread in conservation [6–9,59,60]. For example, subpopulation-scale distribution data are available for <9% of the 1121 IUCN-redlisted mammal species worldwide [19]. The published PVAs did not include ecotourism, but 64 of the 133 species act as ecotourism attractions, and for 20 of these there are also separately published data, quantifiable as population parameters, on both conservation funding effects and ecological impacts of ecotourism. For 11 of these species, the published PVAs omit critical population parameters and are hence unrepeatable. Data to calculate net conservation outcomes of ecotourism are thus available for nine species worldwide (Table 2). Some of these species survive as discrete subpopulations, 21 in all.

**Fig 1:**
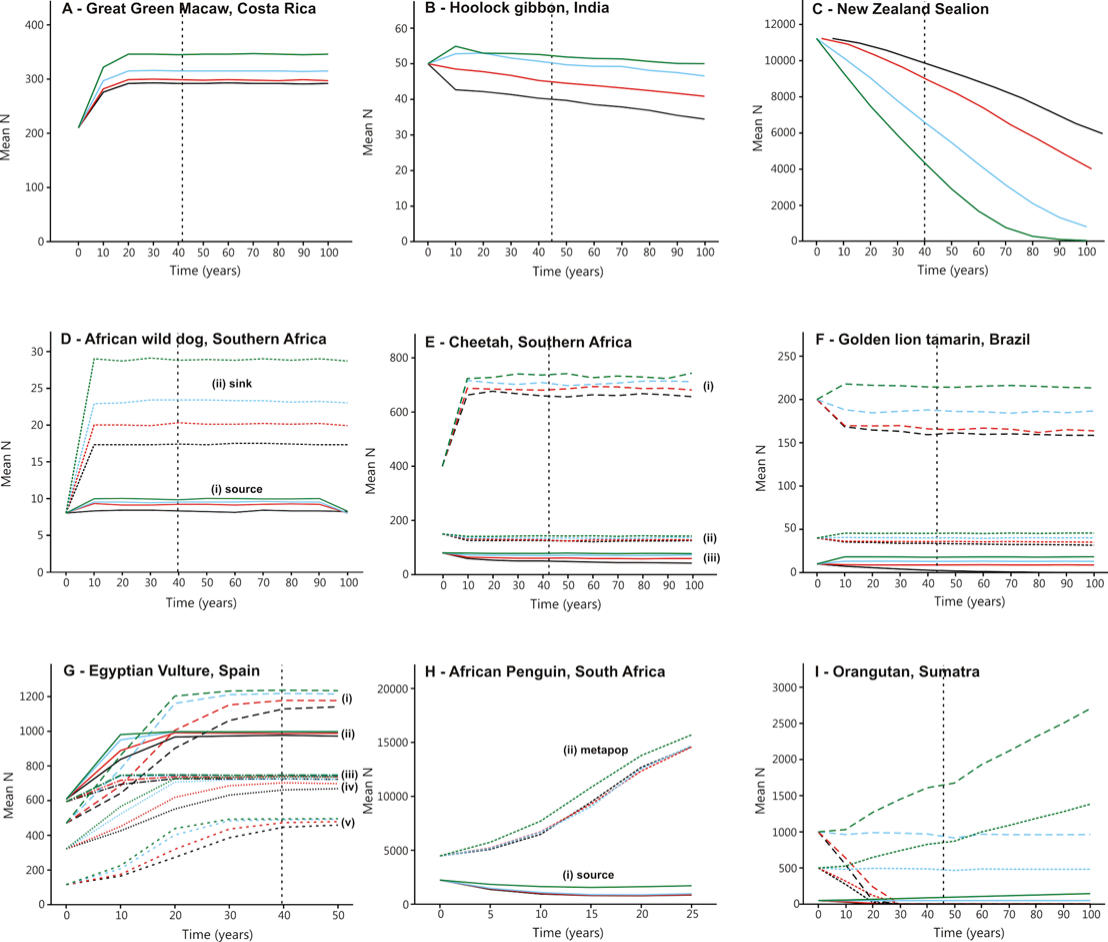
Population trajectories. Population trajectories, incorporating joint net effects of ecotourism at different levels for different species and subpopulations. Timescale: vertical dashed line = 2050. Ecotourism levels: black lines, nil; red, low; blue, medium; green, high. See Tables 1–3 for scientific names, ecotourism parameters and sensitivity analyses; and text for subpopulations and sites.

Population trajectories are shown in Fig 1, and calculation parameters and scientific names in Table 3. Each of the 21 sets of population trajectories represents a different real-world subpopulation of the species concerned. The 4 different levels of ecotourism modelled for each species and subpopulation yield different net outcomes for different subpopulations of different species, depending on the precise mechanisms for ecotourism effects and on the population and habitat parameters. The effectiveness of ecotourism as a conservation tool for threatened species depends on the details of each case.

For great green macaw in Costa Rica, hoolock gibbon in India, and New Zealand sealion (Fig 1A–1C), data are available for only a single subpopulation. For the other six species, data are available for 2–5 distinct subpopulations in different geographical locations, each with its own particular population parameters. For African wild dog (Fig 1D), cheetah (1E), hoolock gibbon, golden lion tamarin in Brazil (1F), great green macaw, and Egyptian vulture in Spain (1G), the net effects of ecotourism on populations are visible within 10–20 years; after that, populations stabilise at levels set by habitat, food and predation.

For some species, notably golden lion tamarin and cheetah, the effects of initial population size outweigh those of ecotourism. For cheetah, ecotourism produces net population increases for a larger subpopulation in a private reserve with high prey density and no predators, even though habitat area is small (Fig 1E i). For two smaller starting populations, ecotourism only reduces the rate of loss: one in a national park with high prey density but also high predator density (1E ii), and the other at low density in an arid environment, with low prey density and significant predation (1E iii).

For hoolock gibbon (Fig 1B) and golden lion tamarin (1F), populations are projected to decrease under zero or low levels of ecotourism, but to increase, at least initially, under moderate or higher levels. For these species, increasing levels of ecotourism yields a consistent net gain at each starting population, even though the starting population size outweighs the effects of ecotourism. The principal ecological mechanism for these two species is through restoration of habitat, increasing effective habitat area available.

For Egyptian vultures (Fig 1G), every subpopulation is projected to increase, stabilising at different levels depending on combinations of population parameters. Subpopulations in the Iberian and Central regions of Spain (Fig 1G ii, iii) behave differently from those in the Cantabrian, Pyrenees and Baetic regions (1E i, iv, v). For this species the effects of ecotourism are consistently positive, and largest at a time-scale of one to two decades, decreasing thereafter.

For African wild dog (Fig 1D), two different subpopulations with the same starting size behave very differently, though both respond positively to ecotourism. One (1D i) is a smaller site-constrained source population, whereas the other (1D ii) is a larger, mortality-constrained sink population. In the case of African penguins (Fig 1H), ecotourism has little effect on a small habitat-limited source subpopulation (1H i), but adds further incremental growth for the overall metapopulation (1H ii), which is increasing in any event.

The most significant effects of ecotourism, from a conservation perspective, occur in the case of orangutan and New Zealand sealion. For three orangutan subpopulations with different starting sizes in Aceh, Sumatra (Fig 1I), numbers are projected to decrease to extinction at zero or low levels of ecotourism, remain stable at moderate levels, and increase at high levels. The net gains from higher levels of ecotourism are large enough to offset the impacts of commercial logging, whereas from lower levels of ecotourism, the gains are insufficient. For orangutan, therefore, different ecotourism intensities make the difference between extinction and survival.

In the case of New Zealand sealion (Fig 1C), populations in the Auckland Islands are declining as a result of fisheries impacts. The net effect of ecotourism is to hasten that decline, with higher levels of ecotourism yielding more severe impacts. The mechanism is that both fisheries and ecotourism increase pup mortality. From fisheries impacts alone, the population is projected to decline by ~45% over the next century. If high-intensity ecotourism is added, it is predicted to become extinct. The net effect of ecotourism is thus opposite to that for orangutan.

## Discussion

The net effects of ecotourism on threatened species may thus be summarized as follows. For those species where ecotourism provides net conservation gains, a variety of different mechanisms are involved: private conservation reserves for cheetah and African wild dog; habitat restoration for hoolock gibbon and golden lion tamarin; reduction in habitat damage for orang-utan; removal of feral predators for African penguin; anti-poaching patrols for great green macaw; and captive breeding and food supplementation for golden lion tamarin, cheetah, and Egyptian vulture.

Different mechanisms also operate for those species where overall population outcomes are negative. For orang-utan, at low ecotourism intensities, positive net ecotourism effects are outweighed by commercial logging. For New Zealand sealion, ecotourism compounds the impacts of intensive fisheries, by increasing pup mortality. For some species, the net effects of ecotourism differ in scale, and sometimes also in direction, for different individual subpopulations.

These analyses thus show that net outcomes of ecotourism for conservation: (i) can be calculated as changes in expected time to extinction for individual threatened species; (ii) may be either positive or negative overall; (iii) differ between species, depending on population parameters and the type and scale of impacts, (iv) operate through different mechanisms for different species and subpopulations; and (v) are affected by other anthropogenic effects occurring simultaneously.

In the longer term, for species under threat from extractive industries such as logging or fisheries, ecotourism can only yield an overall conservation gain if those industries are halted. This has previously been indicated only in qualitative terms [17,22,25]. For threatened species well represented in existing but underfunded conservation reserves, appropriately managed ecotourism can yield an overall net gain in expected survival time. For species poorly represented in conservation reserves and threatened by extractive industries, ecotourism can buy some time whilst other efforts are made to provide protection. Current large-scale international efforts to promote ecotourism as a conservation tool [11,14–16,56–58] therefore deserve support.

More generally, the role of ecotourism in conservation is an example of a complex social-ecological system [2,5,12,13,23] or science-policy interface [61], which requires both natural and social sciences for effective analysis. Each of the species and subpopulations analysed here exists in a different, and changing, social and cultural context. Some are threatened by habitat loss; some are protected by habitat expansion or restoration. Some are threatened by harvesting of individuals, legally or otherwise; some are protected by conservation measures such as captive breeding and translocations which operate at the scale of individuals. Some would survive if left alone, but are threatened by other industry sectors which ecotourism may or may not be able to compete with.

The approach adopted here provides a method to integrate all these effects in ecologically meaningful terms, but it relies on detailed data to derive population parameters. There are many further threatened species subject to ecotourism, for which some but not all the relevant parameters are known. The PVA approach developed here could be applied immediately for these additional species if the missing parameters were available. Research effort to measure such key population parameters for additional threatened species, and to quantify the various effects of ecotourism on these parameters, is now a priority as ecotourism is increasingly advocated as a tool in global conservation.
